# Better understand the semiology of the dysgraphia in DCD children by highlighting a motor control disorder

**DOI:** 10.1192/j.eurpsy.2025.1217

**Published:** 2025-08-26

**Authors:** L. Vaivre-Douret, C. Lopez

**Affiliations:** 1 Department of Medicine Paris Descartes, University of Paris Cité, Faculty of Health; 2 Chair of Neurodevelopmental Clinical Phenotyping, Institut Universitaire de France (IUF); 3 Faculty of Medicine, University of Paris-Saclay, UVSQ, Villejuif, Research team “Neurodevelopment and learning disabilities”, Necker-Enfants Malades hospital, INSERM Unit 1018-CESP; 4 University hospital, AP-HP. Centre, Necker Enfants Malades; 5 Department of Endocrinology, Necker-Enfants Malades hospital, IMAGINE Institute, Paris, France

## Abstract

**Introduction:**

Handwriting is mainly a motor process involving an efficient level of motor organization lead-ing to fine coordination of movements, thus, children with developmental coordination disorder (DCD) are particularly affected with handwriting disorders.

**Objectives:**

We aimed to investigate handwriting disorders in DCD children in order to better understand the semiology of dysgraphia.

**Methods:**

Data from 65 children aged 5 to 15 years (mean age 8.9 years, SD = 2.5) with DCD were collected on DSM-V criteria. They had no other medical or psychiatric condition and born full-term. They were assessed with handwriting testing and standardized assessments of neuropsychological, neurovisual, MRI and neuropsychomotor functions (NP-MOT neurodevelopmental battery), including muscular tone examination. Particular attention was paid to minor neurological dysfunctions (MND) which can be detected with the NP-MOT battery, such as a mild phasic stretch reflex (PSR).

**Results:**

Findings showed a high rate of visual perceptual motor, visuo-spatial, and visuo-constructional impairments (> 82%), visual pursuit disorders (93%) and 89% of handwriting disorders (HD, n=58). Among these HD, there are 83% of poor handwriting (PH) and 17% of dysgraphia (DysG). Moreover, we found in HD, 36% of PSR (with 29% in PH vs 70% in DysG) correlated to imbalance of axial tone (hyper-extension) associated with increase of neuropsychomotor disorders such as dissonance between spontaneous gestural, usual and psychosocial lateralities (P = 0.03), impairments of coordination between upper and lower limbs (P = 0.001), impairments of manual dexterity (P < 0.001), impairments of dynamic balance (P = 0.002), and dysdiadochokinesis (P < 0.001). Comparing PH and DysG groups, dysgraphia is associated to PSR (P = 0.04). 38% of abnormal MRI scans were heterogeneous and non-specific to the level of handwriting disorder and to PSR.

**Conclusions:**

Dysgraphia appears to be a singular disorder as a comorbidity of DCD, which is significantly associated with a high incidence of motor impairments, suggesting a disturbance of the motor pathway (mild distal spasticity of the pyramidal corticospinal tract dysfunction). The presence of MND such as PSR highlights a mild impairment of the motor voluntary movement from the premotor cortex. PH appears primarily due to an immaturity of handwriting gesture consecutive to disorders of coordination programming in DCD.

Dysgraphia should be assessed not only with a simple handwriting test (legibility and speed) but completed with a developmental standardized physical neuropsychomotor examination assessing the presence of MND because to know the nature of the disorder is useful in clinical decision-making processes for handwriting remediation.

**Disclosure of Interest:**

None Declared

